# Supplementary material for: A Comparative Analysis of Selection Pressures Suffered by Mitochondrial Genomes in Two Planthopper Species with Divergent Climate Distributions
Source: Int J Mol Sci. 2023 Nov 28;24(23):16847. doi: 10.3390/ijms242316847 (PMC10706623; doi:10.3390/ijms242316847)
Supplement: Supplementary file 1 [file ijms-24-16847-s001.zip › ijms-2710210-supplementary.pdf]

Table S1 Primer sequences of partial mitochondrial genome amplification in WBPH.

PCR primer sequence (5’–3’)	Product size (bp)
COIF: TTGGTTACTTCCACCCTCCT<br>COIIR: GTTCCACAGATTTCTGAGCACT	1892

Table S2 Primer sequences of complete mitochondrial genome amplification in WBPH.

Fragment	PCR primer sequence (5’–3’)	Product size (bp)
COI - 16S RNA	I-F: TTGGTTACTTCCACCCTCCT<br>I-R: GATACCTTAGGGATAACAGCGT	10912
CYTB-COI	II-F: CTATTATCGGCTGTTCCTTATT<br>II-R: TACTCCTGTTAGACCACCAAT	8429
